# A presentation of iatrogenic hypospadias after traditional circumcision: A case report

**DOI:** 10.1016/j.amsu.2022.104872

**Published:** 2022-11-12

**Authors:** Shukri Said Mohamed, Omar Adam Sheikh, Mesut Kayse Adam, Abdullahi Yusuf Ali, Abdikarim Hussein Mohamed, Ahmed Mead

**Affiliations:** a Department of Pediatric Surgery, Mogadishu Somali Turkey Recep Tayyip Erdoğan Training and Research Hospital, Mogadishu, Somalia; b Faculty of Medicine, Department of Basic Medical Science, Somali National University, Mogadishu, Somalia; c Department of Urology, Mogadishu Somali Turkey Recep Tayyip Erdoğan Training and Research Hospital, Mogadishu, Somalia; d Faculty of Medicine, Department of Basic Medical Science, Salaam University, Mogadishu, Somalia

## Abstract

**Introduction and Importance:**

Traditional circumcisions may cause complications such as hemorrhage, infection, amputations of the penis, meatal stenosis, and urethro-cutaneous fistula. In addition to all these complications, iatrogenic hypospadias, as in our case, is a rare condition. In general, complications are mild and preventable, especially in children, but when the procedure is carried out by unskilled providers, in unsterile conditions, or with inadequate equipment and supplies, severe complications are more likely to occur. Several degrees of urethral erosion, including iatrogenic hypospadias, might result from further injury. Particularly in intensive care facilities, the ventral male urethra can undergo this kind of trauma.

**Case presentation:**

A 4-year-old child was circumcised at the age of 3 years, and after that, he bled profusely. His parents brought him to the hospital after 4 months. On physical examination of the patient, the glans was normal but there was an opening near the glans in the distal urethra at the subcoronal level. After the pre-operative check-up, the patient was prepared for elective surgery. An incision and dissection were performed to reveal the fistula tract all around by placing marker sutures from the edges of the fistula. The fistula opening was repaired with 6/0 PDS (polydioxanone) and a second layer was created over the urethral fistula repair, and then the skin was closed with 4/0 Vicryl (polyglactin).

**Clinical discussion:**

Around the world, circumcision continues to be the most common procedure done on children. Injuries to the penis may actually happen with a 1% complication incidence. A poorly placed suture at the frenulum in an effort to achieve hemostasis is the most frequent cause of the fistula. This causes strangulation and necrosis of a portion of the urethral wall, which leads to the creation of a sub glandular fistula. It is important to properly identify and treat any life-threatening injuries to the urethra as soon as possible.

**Conclusion:**

Considered a medical procedure that necessitates great care, circumcision should only be carried out by qualified surgeons under sterile hospital circumstances. Most circumcision-related injuries result from clamp circumcisions (such as Mogen or Gomco), and they can range from minor loss of penile skin to more serious glans, distal urethral, and penile shaft injuries.

## Highlights

• Hypospadias is the most frequent genital defect after undescended testis and occurs in nearly 1 in 250 newborn males.
• Serious side effects, such as excessive bleeding that results in death, severe urethral injuries, and total or partial amputation of the penis or glans, are possible during or shortly after the surgery.
• The most frequent early (intra-operative) consequences associated with circumcision tend to be minor and curable, including minimal bleeding and edema.

## 1 Introduction

Hypospadias is the most frequent genital defect after undescended testis and occurs in nearly 1 in 250 newborn males and can be combined into one or all three forms: 1. ectopic urethral meatus 2. Penile curvature (chordee) 3. insufficiency of the ventral foreskin [1]. Circumcision is one of the oldest procedures in surgical practice and also one of the most carried out medical procedures today [2]. The most frequent early (intra-operative) consequences associated with circumcision tend to be minor and curable, including minimal bleeding and edema. As with any surgical treatment, complications are possible. According to a study, circumcision consequences can be categorized into five different grades: grade I skin abnormalities, grade II isolated urethral lesions, grade III glans amputations, grade IV corpus cavernosum lesions, and grade V complete loss of the phallus [3]. Serious side effects, such as excessive bleeding that results in death, severe urethral injuries, and total or partial amputation of the penis or glans, are possible during or shortly after the surgery [3]. post-operative problems of circumcision are Pain, wound infection, development of a skin bridge between the penile shaft and the glans, infection, urinary retention, meatal ulcer, meatal stenosis, fistulas, loss of penile sensitivity, sexual dysfunction, and glans penis edema [4]. Most circumcision-related injuries result from clamp circumcisions (such as Mogen or Gomco), and they can range from minor loss of penile skin to more serious glans, distal urethral, and penile shaft injuries [5]. A poorly placed suture at the frenulum in an effort to achieve hemostasis may be the most prevalent cause of a urethrocutaneous fistula after circumcision. Although the exact mechanism of damage is unclear, it is likely that the device's crushing will cause urethral damage. The dorsum of the penis is where the majority of these fistulas open, but they can also be found on the ventral surface [6].

## 2 Case presentation

A 4-year-old child was circumcised at the age of 3 years, and after that, he bled profusely. At that time, he was far away from the hospital, and they decided to wait, and the bleeding stopped spontaneously. His parents noticed the urine wasn't coming out the proper way and they brought him to the hospital after 4 months. At that time, there were no emergency conditions like urinary obstruction, and they came as outpatients of the pediatric surgery department. There is no drug history or other family medical or genetic problems. Their social economy is very poor, and there is no governmental support.

**Fig. 1:**
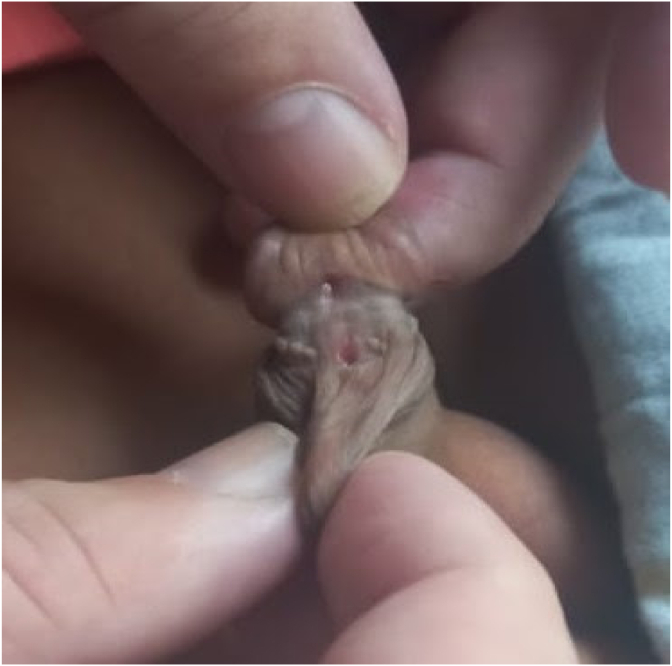
(Subcoronal urethrocutaneous fistula).

**Fig. 2:**
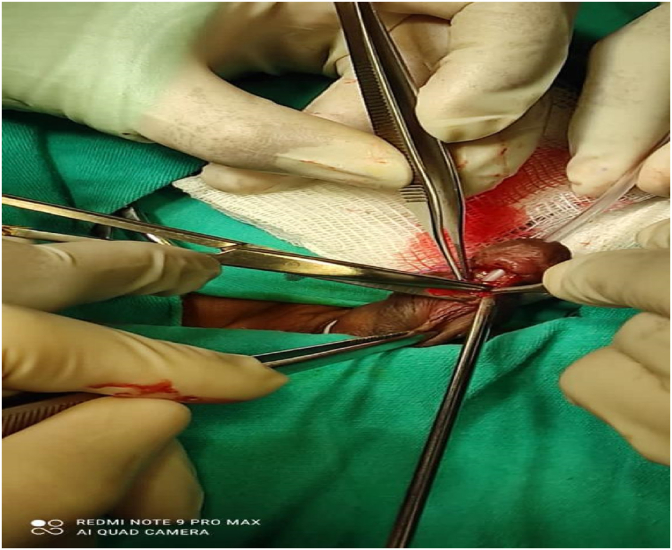
Dissection of mucosa around the fistula.

On physical examination of the patient, the glans was normal. Neither edema nor erythema was detected, but there was an opening near the glans in the distal urethra at the subcoronal level. The catheter advanced freely from the urethral meatus, there was no meatal stenosis or urethral stenosis. The subcoronal urethral fistula was large enough to see the urethral catheter advance from the meatus to the bladder (Fig. 1) (see Fig. 2).

The urine analysis, culture, and other blood tests taken for pre-operative check-ups were normal, and the patient was prepared for elective surgery by a pediatric surgeon. Under general anesthesia with endotracheal intubation, in the decubitus dorsalis position, after sterile staining and covering with povidone-iodine, a 6 French urethral catheter was placed into the bladder from the meatus and fixed with a glans suspension suture. An incision and dissection were performed to reveal the fistula tract all around by placing marker sutures from the edges of the fistula (Picture 2).

**Fig. 3:**
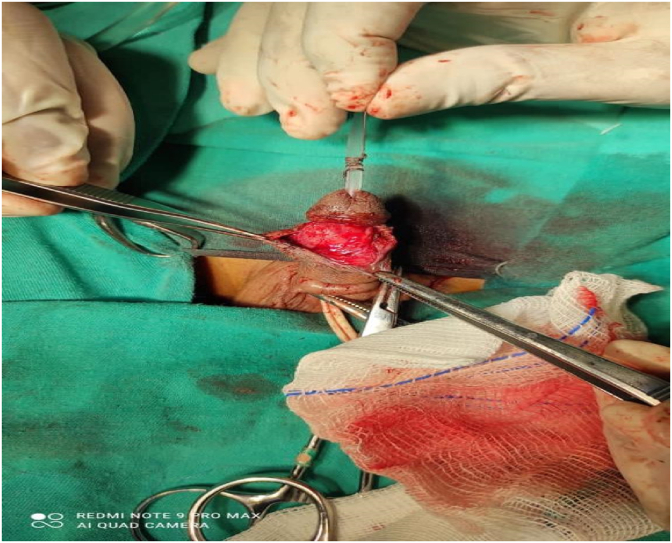
Covering flap over the sutured fistula.

**Fig. 4:**
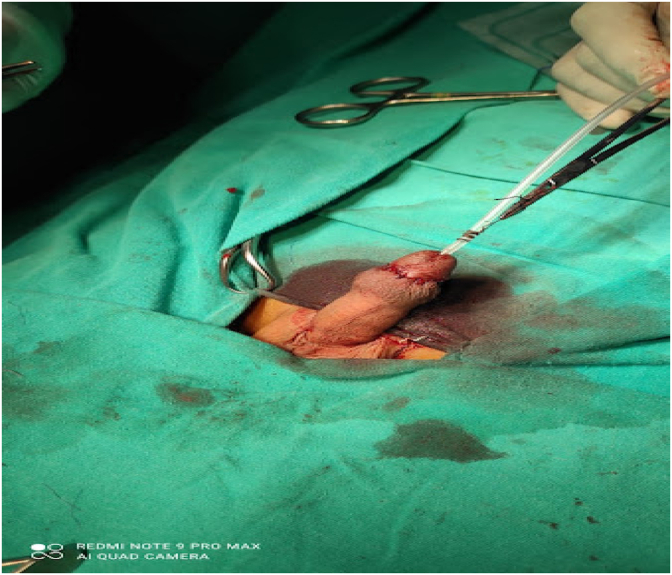
Circumferential suture of the skin.

After free mobilization, the fistula opening was repaired with a 6/0 PDS (polydioxanone) simple suture. In addition, a second layer was created over the urethral fistula repair suture line with a dartos flap (Fig. 3), and then the skin was closed with 4/0 Vicryl (polyglactin) (Fig. 4).

**Fig. 5:**
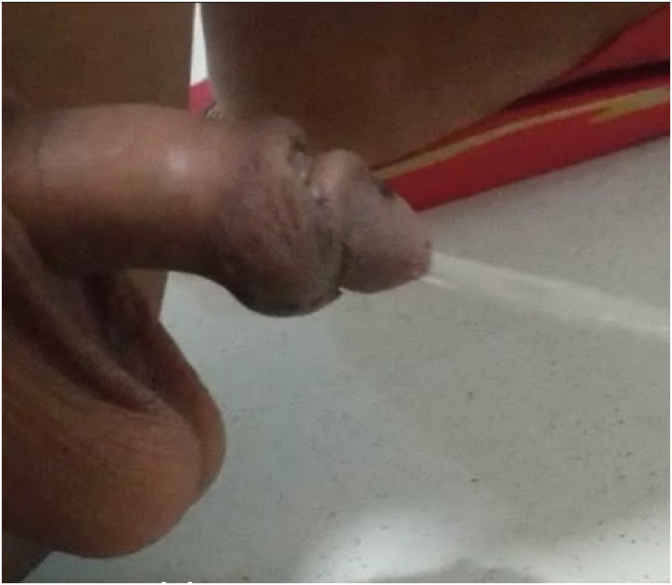
Showing postop micturition.

The urethral catheter was removed on the 7th postoperative day. During voiding, it was tested that the fistula was closed and the large calibrated urine flow was coming from the meatus (Fig. 5). The patient was followed up care every six weeks, and there was no urethral stenosis in the next six months after the operation.

## 3 Discussion

Around the world, circumcision continues to be the most common procedure done on children. Injuries to the penis may actually happen with a 1% complication incidence. Differentiate circumcision fistula with the uncommon congenital urethral fistula, which often develops on the penile shaft and is related to anomalies in the surrounding urethral spongiosum. A circumcision fistula can be successfully corrected in a single stage of surgery [1]. The majority of hypospadias repair procedures involve approximating the limited or severely dissected glans wings on the ventral midline to enclose the neo-urethra and tubularizing the urethra over an 8-fr catheter or stent [7]. A poorly placed suture at the frenulum in an effort to achieve hemostasis is the most frequent cause of the fistula. This causes strangulation and necrosis of a portion of the urethral wall, which leads to the creation of a sub glandular fistula [8]. In our case, he had a history of bleeding while in the process of circumcision and tried to stop it. However, the fistula formed after surgery can occur during surgery or as a result of a deeply positioned frenular suture. It is important to properly identify and treat any life-threatening injuries to the urethra as soon as possible. Once this has been achieved, a precise diagnosis of the source, site, type, and degree of the urethral injury experienced is essential for effective prompt care (within 48 h) [9]. After stabilizing the patient, definite care of urethral damage can begin once the diagnosis has been made [9]. Unfortunately, our case came late (4 months after the circumcision) because of low socioeconomic conditions and loss of awareness. A retrospective study from Turkey of 407 boys who had been circumcised at two traditional mass circumcision events found a significant incidence of complications; circumcision by non-medically educated workers is typically followed by a high rate of both early and late complications [10]. Boys' genitalia can be carefully examined when they are admitted to a hospital for circumcision, but the most crucial objective is to improve the public's view of the surgery, which is typically misunderstood [3]. We recommend educating all inexperienced makers, and the government must control non-medical privet workers to decrease the complications of circumcision.

## 4 Conclusion

Considered a medical procedure that necessitates great care, circumcision should only be carried out by qualified surgeons under sterile hospital circumstances. Otherwise, complications during or soon after the procedure may occur, including serious urethral injuries, life-threatening profuse bleeding that leads to death, and whole or partial amputation of the penis or glans. The most frequent cause of a urethral fistula following circumcision may be a poorly placed suture at the frenulum made in an effort to achieve hemostasis.

## Ethical approval

An institution board of review is not required ethics committee approval for the case reports.

## Please state any sources of funding for your research

We declare that we have no funding source.

## Author contribution

SS Mohamed: Idea of the research, writing of the manuscript, Final revision of the data, and intellectual content related to pediatric surgery.

OA Sheikh: Idea of the research, Review of data, writing the paper.

MK Adam: Review of the research, writing conclusion, and intellectual content related to pediatric surgery.

AY Ali: data collection, Review of data, and intellectual content related to pediatric surgery.

AH Mohamed: Review of data and grammatical correction.

A Mead: Review of the research.

## Registration of research studies

1. Name of the registry: **Not applicable**
2. Unique Identifying number or registration ID: **Not applicable**
3. Hyperlink to your specific registration (must be publicly accessible and will be checked): **Not applicable**

## Guarantor

As Corresponding Author, I confirmed that the manuscript has been read and approved by all named authors.

## Consent

Written informed consent was obtained from the patient's parent for publication of this case report and accompanying images. A copy of the written consent is available for review by the Editor-in-Chief of this journal on request.

## Declaration of competing interest

No conflicts of interest in this work.
